# Robust Position Control of an Over-actuated Underwater Vehicle under Model Uncertainties and Ocean Current Effects Using Dynamic Sliding Mode Surface and Optimal Allocation Control

**DOI:** 10.3390/s21030747

**Published:** 2021-01-22

**Authors:** Mai The Vu, Tat-Hien Le, Ha Le Nhu Ngoc Thanh, Tuan-Tu Huynh, Mien Van, Quoc-Dong Hoang, Ton Duc Do

**Affiliations:** 1 School of Intelligent Mechatronics Engineering, Sejong University, 98 Gunja-dong, Gwangjin-gu, Seoul 143-747, Korea; 2 Department of Naval Architecture and Marine System Engineering, Ho Chi Minh City University of Technology (HCMUT), 268 Ly Thuong Kiet Street, District 10, Ho Chi Minh City 700000, Vietnam; 3 Vietnam National University Ho Chi Minh City (VNU-HCM), Linh Trung Ward, Thu Duc District, Ho Chi Minh City 700000, Vietnam; 4 HUTECH Institute of Engineering, Ho Chi Minh City University of Technology (HUTECH), Ho Chi Minh City 700000, Vietnam; hlnn.thanh@hutech.edu.vn; 5 Department of Electrical Engineering, Yuan Ze University, No. 135, Yuandong Road, Zhongli 320, Taoyuan 32003, Taiwan; huynhtuantu@saturn.yzu.edu.tw; 6 Department of Electrical Electronic and Mechanical Engineering, Lac Hong University, No. 10, Huynh Van Nghe Road, Bien Hoa, Dong Nai 830000, Vietnam; 7 School of Electronics, Electrical Engineering and Computer Science, Queen’s University, Belfast BT7 1NN, UK; m.van@qub.ac.uk; 8 Institute of Mechanical Engineering, Vietnam Maritime University, 484 Lachtray Street, Hai Phong City 182582, Vietnam; hoangquocdong.vimaru@gmail.com; 9 Department of Mechanical Engineering, Kyung Hee University, Seoul 130-701, Korea; 10 Department of Robotics and Mechatronics, School Engineering and Digital Sciences, Nazarbayev University, Nur-Sultan Z05H0P9, Kazakhstan; doduc.ton@nu.edu.kz

**Keywords:** dynamic sliding mode controller, least-squares method, position control, quadratic programming, underwater vehicle

## Abstract

Underwater vehicles (UVs) are subjected to various environmental disturbances due to ocean currents, propulsion systems, and un-modeled disturbances. In practice, it is very challenging to design a control system to maintain UVs stayed at the desired static position permanently under these conditions. Therefore, in this study, a nonlinear dynamics and robust positioning control of the over-actuated autonomous underwater vehicle (AUV) under the effects of ocean current and model uncertainties are presented. First, a motion equation of the over-actuated AUV under the effects of ocean current disturbances is established, and a trajectory generation of the over-actuated AUV heading angle is constructed based on the line of sight (LOS) algorithm. Second, a dynamic positioning (DP) control system based on motion control and an allocation control is proposed. For this, motion control of the over-actuated AUV based on the dynamic sliding mode control (DSMC) theory is adopted to improve the system robustness under the effects of the ocean current and model uncertainties. In addition, the stability of the system is proved based on Lyapunov criteria. Then, using the generalized forces generated from the motion control module, two different methods for optimal allocation control module: the least square (LS) method and quadratic programming (QP) method are developed to distribute a proper thrust to each thruster of the over-actuated AUV. Simulation studies are conducted to examine the effectiveness and robustness of the proposed DP controller. The results show that the proposed DP controller using the QP algorithm provides higher stability with smaller steady-state error and stronger robustness.

## 1. Introduction

The ocean covers approximately 70% of the Earth’s surface and provides us many natural and mineral resources. Moreover, resources on land are being steadily depleted, and thus, exploring the resources in the ocean such as oil, gas, and minerals under the seabed has been increasingly focused in recent years. However, it is difficult to explore and investigate very wide underwater environments in usual ways using manned systems and human divers. As a result, the UVs, especially unmanned systems that can carry out difficult missions without risking human lives, become popular at the moment.

At present, the UVs are utilized in a variety of applications such as scientific surveying, underwater surveillance, oceanographic research, environment monitoring, natural resource exploration, subsea structure inspection and maintenance, anti-submarine warfare, mine-field operation, and industrial fields, etc. [1,2,3,4,5]. Such UVs are often classified into two types—remotely operated vehicles (ROVs) [6,7] and autonomous underwater vehicles (AUVs) [8,9]. Recently, several studies for the ROV have been conducted by many engineers and researchers with a number of different designs proposed [10,11,12,13,14].

The UV is one of the intelligent motion platforms, which can navigate autonomously and safely in the real marine environment and complete many challenging tasks, especially for the navy and marine industries [15,16]. However, they are a high and coupled nonlinear system, which preserves model-uncertainties, time-varying dynamic model and are strongly affected by external disturbances such as the ocean current, wave, un-predicted underwater currents, and so on [17,18]. To handle the uncertainties and disturbances and improve the tracking performances of the UVs, many control techniques have been developed for UVs over the past few years. Among them are linear controllers [19,20], fuzzy logic control [21,22], SMC controllers [23,24], predictive control algorithms [25,26], and neural network control strategies [27], and so on.

In general, to perform a wider range of missions in the sea, the UVs are usually equipped with multiple thrusters. While it provides redundant thrusters for the UVs, this designed configuration makes the UVs face another challenging problem is that the solution to the thrust allocation problem is not unique. Therefore, the DP controller must resolve the thruster redundancy of the UV. The DP technologies play an important role in ocean research and various applications of mobile ocean robots. Moreover, the significant development of ocean robotics has extended the range of applications of DP control, which is mostly applied to unmanned underwater vehicles (UUVs) and unmanned surface vehicles (USVs). The DP control systems are used to control the linear position and heading angle of the marine vehicles against environmental disturbances using their thrusters. In recent decades, various DP control techniques have been proposed. These techniques include PID controller [28], adaptive control [29], SMC control [30], fuzzy control [31], neural network control [32], and so on. Although the above controllers are convenient to be implemented into the physical UVs due to their simple structure, they show less capacity to solve the thruster saturation problem in the DP control design. However, the thruster saturation phenomenon always occurs in practice because of the physical limitations of the propulsion system.

Evidently, the marine vehicles in the sea are affected by various forces and moments such as the waves, ocean currents, propulsion systems, and uncertainties model. Practically, due to the effects of the complex environmental disturbances, it is almost impossible to keep a UV stayed permanently at the desired static position and the desired heading angle. Motivated by the above challenging issues, this paper investigates a trajectory generation and position control for a hovering AUV with four horizontal and three vertical thrusters taking the effects of the model uncertainties and the ocean current into consideration. The DP controller, which consists of motion control and an allocation control strategy, is used to control the thrust of the seven thrusters to obtain an optimal adjustment of the linear position and the heading angle of the UV. First, the motion control in the DP system is designed using a DSMC law to suppress the external disturbances imposing on the AUV. Then, in order to handle the allocation control problem in the DP system, two candidate allocation controllers: the LS and QP method, are designed and compared. Finally, a numerical simulation is carried out to observe and analyze the effects of the ocean current on the motion of the AUV. In addition, to eliminate the ocean current effects on the AUV, the second simulation is implemented with the DP controllers using both suggested optimal allocation control strategies for evaluating the algorithm. The simulation results demonstrate that the QP method of the optimal allocation control module is the best solution in terms of offering a faster transient response and lower steady-state error.

This paper is constructed as follows: Section 2 formulates the kinematic and kinetic models of the AUV with the ocean current disturbances. Section 3 presents the trajectory generation of the heading angle of the AUV using the LOS guidance. Then, Section 4 describes the DP control system of the AUV, which consists of two cascade control modules: the motion control law and the allocation control module. In this section, the motion control is designed using a robust DSMC to eliminate the ocean current effects and the model uncertainties. In addition, two optimal algorithms for the allocation control module, i.e., the LM method and the QP method, are also proposed. Next, Section 5 provides some numerical simulation results and discussions using the established AUV model and the designed DP control system. Finally, Section 6 presents the conclusions of this paper.

## 2. Mathematical Model of an over-Actuated AUV under the Ocean Current Effects

### 2.1. Coordinate System

**Figure 1:**
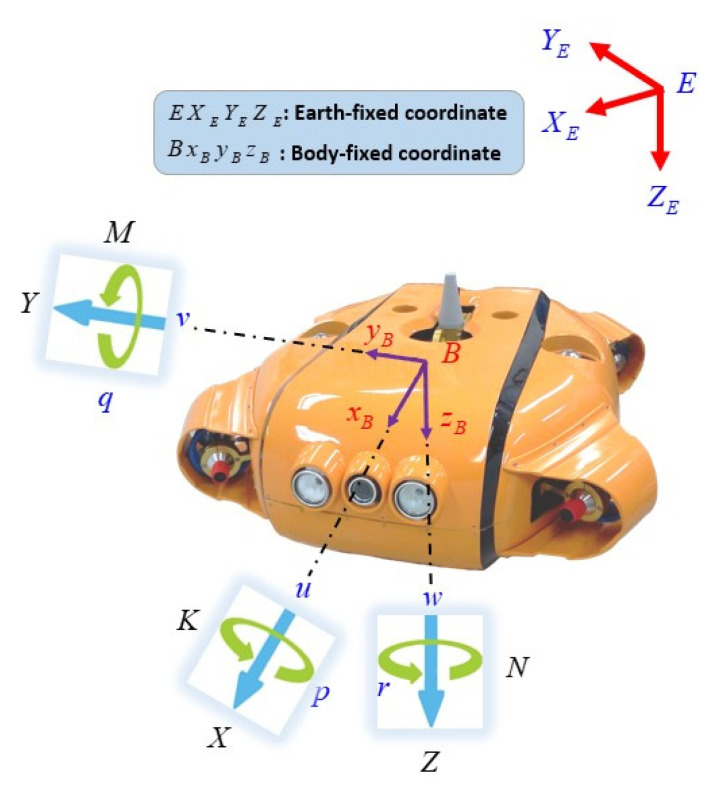
Coordinate system of an autonomous underwater vehicle (AUV).

The modeling of the AUV involves the study of the kinematic and kinetic models. First, we provide the coordinate system of the AUV and definitions of its motion parameters to obtain the six-DOF nonlinear dynamics model of the AUV. The model description of the AUV is based on two reference coordinate systems, as shown in Figure 1.

- The body-fixed (BF) frame is attached to the center of gravity of the AUV: B-XYZ;
- The Earth-fixed (EF) frame system which can be taken as linked to the Earth in the case of the AUV moving at slow speed:$E-X_{E}Y_{E}Z_{E}$.

The AUV model that used in this paper is the hovering over-actuated type AUV with four horizontal thrusters and three vertical thrusters. The six-DOF equation of the motion is used whose state vectors are represented as [33]:

$\eta ={\left[\begin{array}{cc} \eta_{1}^{T} & \eta_{2}^{T} \end{array}\right]}^{T}$: denotes the linear positions and Euler angles vector of the AUV in the EF frame $E-X_{E}Y_{E}Z_{E}$, describing the linear position $\eta_{1}={\left[x,y,z\right]}^{T}$ and the angular position $\eta_{2}={\left[\phi ,\theta ,\psi \right]}^{T}$, where x, y, z are the linear position of the AUV while, ϕ, θ, and ψ are three Euler angles: roll, pitch and yaw, respectively.

$\nu ={\left[\begin{array}{cc} \nu_{1}^{T} & \nu_{2}^{T} \end{array}\right]}^{T}$: represents the velocities vector in the BF frame B-XYZ, describing the linear velocity $\nu_{1}={\left[u,v,w\right]}^{T}$ and the angular velocity $v_{2}={\left[p,q,r\right]}^{T}$.

### 2.2. Kinematic Equations

Because we use two different coordinate systems in the AUV model, a coordinate transformation matrix is used to change the representation of the motion of the AUV from the BF frame to the EF frame or vice versa is needed. Thus, the kinematic equations for an AUV are given as:

(1) $${\dot{\eta }}_{1}=J_{1}(\eta_{2})\nu_{1}$$

(2) $${\dot{\eta }}_{2}=J_{2}(\eta_{2})\nu_{2}$$

in which

(3) $$J_{1}\left(\eta_{2}\right)=\left[\begin{array}{ccc} c\psi c\theta & -s\psi c\phi +s\phi s\theta c\psi & s\psi s\phi +s\theta c\psi c\phi \\ s\psi c\theta & c\psi c\phi +s\phi s\theta s\psi & -c\psi s\phi +s\theta s\psi c\phi \\ -s\theta & s\phi c\theta & c\phi c\theta \end{array}\right]$$

(4) $$J_{2}\left(\eta_{2}\right)=\left[\begin{array}{ccc} 1 & s\phi t\theta & c\phi t\theta \\ 0 & c\phi & -s\phi \\ 0 & s\phi /c\theta & c\phi /c\theta \end{array}\right]$$

where s(.), c(.) and t(.) are short notations for sin (.), cos (.) and tan (.), respectively.

### 2.3. Kinetic Equations

Generally, the motion of the AUV is presented as the six-DOF nonlinear equation. In this section, the nonlinear dynamic equation of the AUV, which expressed in the BF frame, can be formulated in matrix form [34] as:

(5) $$M\dot{v}+C(v)v+D(v)v+G(\eta )=\tau +\tau_{d}$$

where,

*M*: Inertial matrix;

*C*(*v*): Coriolis and centripetal matrix;

*D*(*v*): Damping matrix;

G(η): Matrix of restoring force and moments;

τ: Thruster forces and moments;

$\tau_{d}$: External disturbance forces and moments.

#### 2.3.1. Inertial Matrix

The inertial matrix is described as the sum of an inertial matrix of the AUV itself $M_{RB}$ and a hydrodynamic additional inertial matrix $M_{A}$ due to the inertial of the surrounding fluid.

(6) $$M=M_{RB}+M_{A}\in R^{6\times 6}$$

(7) $$M_{RB}\;=\left[\begin{array}{cccccc} m & 0 & 0 & 0 & mz_{G} & -my_{G}\\ 0 & m & 0 & -mz_{G} & 0 & mx_{G}\\ 0 & 0 & m & my_{G} & -mx_{G} & 0\\ 0 & -mz_{G} & my_{G} & I_{xx} & I_{xy} & I_{xz}\\ mz_{G} & 0 & -mx_{G} & I_{yx} & I_{yy} & I_{yz}\\ -my_{G} & mx_{G} & 0 & I_{zx} & I_{zy} & I_{zz} \end{array}\right]$$

(8) $$M_{A}=\left[\begin{array}{cccccc} X_{\dot{u}} & 0 & 0 & 0 & 0 & 0\\ 0 & Y_{\dot{v}} & 0 & 0 & 0 & Y_{\dot{r}}\\ 0 & 0 & Z_{\dot{w}} & 0 & Z_{\dot{q}} & 0\\ 0 & 0 & 0 & K_{\dot{p}} & 0 & 0\\ 0 & 0 & M_{\dot{w}} & 0 & M_{\dot{q}} & 0\\ 0 & N_{\dot{v}} & 0 & 0 & 0 & N_{\dot{r}} \end{array}\right]$$

where *m* denotes the mass of the AUV; *x_G_*, *y_G_*, and *z_G_* denote the mass center of the AUV, and *I_ij_* is the inertia tensor for each axis of subscripts.

#### 2.3.2. Coriolis and Centripetal Matrix

The Coriolis and centripetal matrix are defined as the sum of a rigid-body Coriolis and centripetal matrix of the AUV $C_{RB}(v)$ and an added-mass Coriolis and centripetal matrix $C_{A}(v)$.

(9) $$C(v)=C_{RB}(v)+C_{A}(v)\in R^{6\times 6}$$

(10) $$\begin{array}{l} C_{RB}(v)=[\begin{array}{ccc} 0 & 0 & 0\\ 0 & 0 & 0\\ 0 & 0 & 0\\ -m\left(y_{G}q+z_{G}r\right) & m\left(y_{G}q+w\right) & m\left(z_{G}p-v\right)\\ m\left(x_{G}q-w\right) & -m\left(z_{G}r+x_{G}p\right) & m\left(z_{G}q+u\right)\\ m\left(x_{G}r+v\right) & m\left(y_{G}r-u\right) & -m\left(x_{G}p+y_{G}q\right) \end{array}\\ \;\;\;\;\;\begin{array}{ccc} m\left(y_{G}q+z_{G}r\right) & -m\left(x_{G}q-w\right) & -m\left(x_{G}r+v\right)\\ -m\left(y_{G}q+w\right) & m\left(z_{G}r+x_{G}p\right) & -m\left(y_{G}r-u\right)\\ -m\left(z_{G}p-v\right) & -m\left(z_{G}q+u\right) & m\left(x_{G}p+y_{G}q\right)\\ 0 & -I_{yz}q-I_{xz}p+I_{zz}r & I_{yz}r+I_{xy}p-I_{yy}q\\ I_{yz}q+I_{xz}p-I_{zz}r & 0 & -I_{xz}r-I_{xy}q+I_{xx}p\\ -I_{yz}r-I_{xy}p+I_{yy}q & I_{xz}r+I_{xy}q-I_{xx}p & 0 \end{array}] \end{array}$$

(11) $$C_{A}(v)=-\left[\begin{array}{cccccc} 0 & 0 & 0 & 0 & -Z_{\dot{w}}w & Y_{\dot{v}}v\\ 0 & 0 & 0 & Z_{\dot{w}}w & 0 & -X_{\dot{u}}u\\ 0 & 0 & 0 & -Y_{\dot{v}}v & X_{\dot{u}}u & 0\\ 0 & -Z_{\dot{w}}w & Y_{\dot{v}}v & 0 & -N_{\dot{r}}r & M_{\dot{q}}q\\ Z_{\dot{w}}w & 0 & -X_{\dot{u}}u & N_{\dot{r}}r & 0 & -K_{\dot{p}}p\\ -Y_{\dot{v}}v & X_{\dot{u}}u & 0 & -M_{\dot{q}}q & K_{\dot{p}}p & 0 \end{array}\right]$$

#### 2.3.3. Damping Matrix

The damping matrix of the AUV in the fluid $D(v)\in R^{6\times 6}$, which consists of the force and the moment of the first and second-order of the velocities, can be represented as:

(12) $$D(v)=-diag\{X_{u},Y_{v},Z_{w},K_{p},M_{q},N_{r}\}-diag\{X_{u\left|u\right|}\left|u\right|,Y_{v\left|v\right|}\left|v\right|,Z_{w\left|w\right|}\left|w\right|,K_{p\left|p\right|}\left|p\right|,M_{q\left|q\right|}\left|q\right|,N_{r\left|r\right|}\left|r\right|\}$$

The values of the damping matrix components are given from field tests.

#### 2.3.4. Restoring Forces and Moments

By assuming that the center of the buoyancy of the AUV expressed in the BF frame is ${\left[x_{b},0,z_{b}\right]}^{T}$, the restoring forces and moments $G(\eta )\in R^{6\times 1}$ can be defined as:

(13) $$G(\eta )=\left[\begin{array}{c} \left(W-B\right)\sin\theta \\ -\left(W-B\right)\cos\theta \sin\phi \\ -\left(W-B\right)\cos\theta \cos\phi \\ -\left(y_{G}W-y_{b}B\right)\cos\theta \cos\phi +(z_{G}W-z_{b}B)\cos\theta \sin\phi \\ (z_{G}W-z_{b}B)\sin\theta +(x_{G}W-x_{b}B)\cos\theta \cos\phi \\ -(x_{G}W-x_{b}B)\cos\theta \sin\phi -\left(y_{G}W-y_{b}B\right)\sin\theta \end{array}\right]$$

where *W* and *B* are the force of gravity and the force of buoyancy, respectively.

Furthermore, all the hydrodynamic coefficients used in the above equations are given in Table 1.

### 2.4. Thruster Configuration Matrix

**Figure 2:**
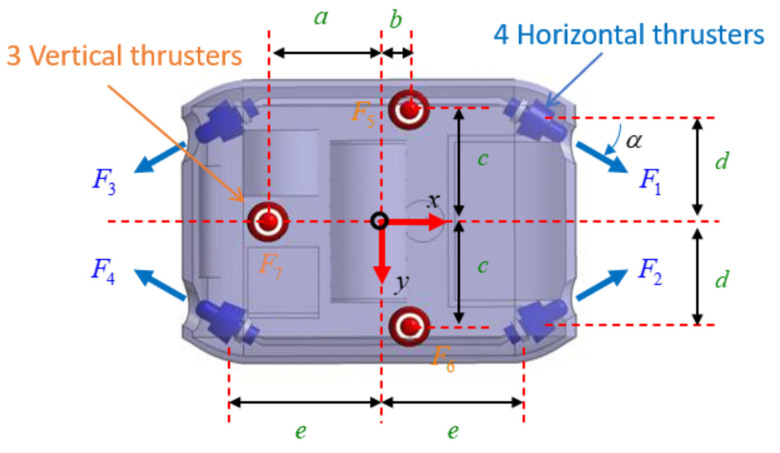
Thruster arrangement of the AUV.

In this paper, an over-actuated AUV is used to define the thruster configuration matrix, as shown in Figure 2. It can be seen that the AUV uses four horizontal thrusters to control the surge, sway, and yaw motions of the AUV, while its three vertical thrusters are applied for heaving, pitching, and rolling motion. Moreover, we assume ${\left(x_{i},y_{i},z_{i}\right)}_{i=1\ldots 7}$ is the center of the *i*-th thruster, and the angle between the longitudinal axis and the direction of the thruster force is $\alpha =30^{0}$.

Since four horizontal thrusters are located at the bow and the stern part, the moment in the horizontal plane caused by these thrusters $T_{i=1,2,3,4}$ can be calculated as:

(14) $${\vec{r}}_{1}\times {\vec{F}}_{1}=\left[\begin{array}{c} x_{1}\\ y_{1}\\ z_{1} \end{array}\right]\times \left[\begin{array}{c} F_{1}\cos\alpha \\ F_{1}\sin\alpha \\ 0 \end{array}\right]=\left[\begin{array}{c} e\\ -d\\ 0 \end{array}\right]\times \left[\begin{array}{c} F_{1}\cos\alpha \\ F_{1}\sin\alpha \\ 0 \end{array}\right]=(eF_{1}\sin\alpha +dF_{1}\cos\alpha )\vec{k}$$

(15) $${\vec{r}}_{2}\times {\vec{F}}_{2}=\left[\begin{array}{c} x_{2}\\ y_{2}\\ z_{2} \end{array}\right]\times \left[\begin{array}{c} F_{2}\cos\alpha \\ -F_{2}\sin\alpha \\ 0 \end{array}\right]=\left[\begin{array}{c} e\\ d\\ 0 \end{array}\right]\times \left[\begin{array}{c} F_{2}\cos\alpha \\ -F_{2}\sin\alpha \\ 0 \end{array}\right]=(-eF_{2}\sin\alpha -dF_{2}\cos\alpha )\vec{k}$$

(16) $${\vec{r}}_{3}\times {\vec{F}}_{3}=\left[\begin{array}{c} x_{3}\\ y_{3}\\ z_{3} \end{array}\right]\times \left[\begin{array}{c} -F_{3}\cos\alpha \\ F_{3}\sin\alpha \\ 0 \end{array}\right]=\left[\begin{array}{c} -e\\ -d\\ 0 \end{array}\right]\times \left[\begin{array}{c} -F_{3}\cos\alpha \\ F_{3}\sin\alpha \\ 0 \end{array}\right]=(-eF_{3}\sin\alpha -dF_{3}\cos\alpha )\vec{k}$$

(17) $${\vec{r}}_{4}\times {\vec{F}}_{4}=\left[\begin{array}{c} x_{4}\\ y_{4}\\ z_{4} \end{array}\right]\times \left[\begin{array}{c} -F_{4}\cos\alpha \\ -F_{4}\sin\alpha \\ 0 \end{array}\right]=\left[\begin{array}{c} -e\\ d\\ 0 \end{array}\right]\times \left[\begin{array}{c} -F_{4}\cos\alpha \\ -F_{4}\sin\alpha \\ 0 \end{array}\right]=(-eF_{4}\sin\alpha +dF_{4}\cos\alpha )\vec{k}$$

Similar to the horizontal plane, the moment induced by three vertical thrusters $T_{i=5,6,7}$ can also be computed as:

(18) $${\vec{r}}_{5}\times {\vec{F}}_{5}=\left[\begin{array}{c} x_{c5}\\ y_{c5}\\ z_{c5} \end{array}\right]\times \left[\begin{array}{c} 0\\ 0\\ F_{5} \end{array}\right]=\left[\begin{array}{c} b\\ -c\\ 0 \end{array}\right]\times \left[\begin{array}{c} 0\\ 0\\ F_{5} \end{array}\right]=(-cF_{5})\vec{i}-(bF_{5})\vec{j}$$

(19) $${\vec{r}}_{6}\times {\vec{F}}_{6}=\left[\begin{array}{c} x_{c6}\\ y_{c6}\\ z_{c6} \end{array}\right]\times \left[\begin{array}{c} 0\\ 0\\ F_{6} \end{array}\right]=\left[\begin{array}{c} b\\ c\\ 0 \end{array}\right]\times \left[\begin{array}{c} 0\\ 0\\ F_{6} \end{array}\right]=(cF_{6})\vec{i}-(bF_{6})\vec{j}$$

(20) $${\vec{r}}_{7}\times {\vec{F}}_{7}=\left[\begin{array}{c} x_{c7}\\ y_{c7}\\ z_{c7} \end{array}\right]\times \left[\begin{array}{c} 0\\ 0\\ F_{7} \end{array}\right]=\left[\begin{array}{c} -a\\ 0\\ 0 \end{array}\right]\times \left[\begin{array}{c} 0\\ 0\\ F_{7} \end{array}\right]=(0F_{7})\vec{i}+(aF_{7})\vec{j}$$

As a result, the generalized forces and moments created by all thrusters can be expressed by:

(21) $$\begin{array}{l} F_{thrust}=F_{Tx}\vec{i}+F_{Ty}\vec{j}+F_{Tz}\vec{k}\\ =(F_{1}+F_{2}-F_{3}-F_{4})\cos\alpha \vec{i}+(F_{1}-F_{2}+F_{3}-F_{4})\sin\alpha \vec{j}+(F_{5}+F_{6}+F_{7})\vec{k} \end{array}$$

(22) $$\begin{array}{l} M_{thrust}=M_{Tx}\vec{i}+M_{Ty}\vec{j}+M_{Tz}\vec{k}\\ =(-cF_{5}+cF_{6}+0F_{7})\vec{i}+(-bF_{5}-bF_{6}+aF_{7})\vec{j}+(eF_{1}\sin\alpha +dF_{1}\cos\alpha \\ -eF_{2}\sin\alpha -dF_{2}\cos\alpha -eF_{3}\sin\alpha -dF_{3}\cos\alpha +eF_{4}\sin\alpha +dF_{4}\cos\alpha )\vec{k} \end{array}$$

Alternatively, the thruster allocation can be conducted in the matrix form as:

(23) $$U_{v}=LF$$

(24) $$U_{v}={\left[\begin{array}{cccccc} F_{Tx} & F_{Ty} & F_{Tz} & M_{Tx} & M_{Ty} & M_{Tz} \end{array}\right]}^{T}$$

(25) $$F={\left[\begin{array}{ccccccc} F_{1} & F_{2} & F_{3} & F_{4} & F_{5} & F_{6} & F_{7} \end{array}\right]}^{T}$$

(26) $$L=\left[\begin{array}{ccccccc} c\alpha & c\alpha & -c\alpha & -c\alpha & 0 & 0 & 0\\ s\alpha & -s\alpha & s\alpha & -s\alpha & 0 & 0 & 0\\ 0 & 0 & 0 & 0 & 1 & 1 & 1\\ 0 & 0 & 0 & 0 & -c & c & 0\\ 0 & 0 & 0 & 0 & -b & b & a\\ dc\alpha +es\alpha & -dc\alpha -es\alpha & -dc\alpha -es\alpha & dc\alpha +es\alpha & 0 & 0 & 0 \end{array}\right]$$

where cα is cos(α), sα is sin(α); $U_{v}$, *F* and *L* are the vectors of the generalized forces and moments generated by the seven thrusters, the vector of the seven thruster forces, and the thruster configuration matrix, respectively.

### 2.5. Dynamic Model of the Over-actuated AUV Including Ocean Current Effects

The dynamic model of the AUV in Equation (5) is obtained without considering the ocean current effects. The influence of the ocean current on the motions of the AUV is significant; thus, it is necessary to analyze the response of the AUV to environmental disturbances. To observe the influence of the ocean currents on the AUV, some simplifications are first, made as:

- As the AUV is a submerged object, the wave-induced currents are quite negligible;
- The ocean current is slowly varying or constant, and its speed is bounded in the specified range;
- The equations of the motions can be expressed in terms of the relative velocity between the AUV and the ocean currents.

Since the ocean current is a complex and irregular form, it is difficult to model and to consider its effects on the AUV. Based on the Gauss–Markov process [35], the ocean current velocity is modeled as:

(27) $$\dot{\xi }(t)=-a\xi (t)+W_{c}(t)$$

where,

(28) $$\xi (t)=\left[\begin{array}{c} V_{c}(t)\\ \alpha (t)\\ \beta (t) \end{array}\right],a=\left[\begin{array}{ccc} a_{1} & 0 & 0\\ 0 & a_{2} & 0\\ 0 & 0 & a_{3} \end{array}\right],W_{c}(t)=\left[\begin{array}{c} w_{1}(t)\\ w_{2}(t)\\ w_{3}(t) \end{array}\right]$$

in which $W_{c}\in R^{3\times 1}$ is Gaussian white noise, and $a\in R^{3\times 3}>0$ is a suitable constant matrix, and $\xi (t)\in R^{3x1}$ is the variable vector of the ocean current model that has three components such as ocean current speed in the fluid frame $V_{c}(t)$, the angle of attack α(t), and the sideslip angle β(t).

Now, the ocean current speed is bounded in the form as:

(29) $$\xi_{\min}\leq \xi (t)\leq \xi_{\max}$$

Assuming that the fluid is irrotational, the six components of the ocean current speed vector in the EF frame is expressed as:

(30) $$V_{c}^{E}={\left[\begin{array}{cccccc} v_{x} & v_{y} & v_{z} & 0 & 0 & 0 \end{array}\right]}^{T}$$

where the linear components of the ocean current in the three axes of the EF frame can be defined as:

(31) $$v_{x}=V_{c}\cos\alpha \cos\beta$$

(32) $$v_{y}=V_{c}\sin\beta$$

(33) $$v_{z}=V_{c}\sin\alpha \cos\beta$$

Using the rotation transformation expressed in Equation (3), the ocean current speed in the BF frame is defined as:

(34) $$V_{c}^{B}={\left[\begin{array}{cccccc} u_{c}^{B} & \upsilon_{c}^{B} & w_{c}^{B} & 0 & 0 & 0 \end{array}\right]}^{T}=diag\left[R^{T}(\eta ),0_{3\times 3}\right]V_{c}^{E}$$

where, $u_{c}^{B}$, $\upsilon_{c}^{B}$ and $w_{c}^{B}$ represent the ocean current speeds in the surge and sway, and heave motions of the AUV, respectively.

Consequently, the motion of the AUV can be described in term of the relative speed as:

(35) $$v_{r}=v-V_{c}^{B}={\left[\begin{array}{cccccc} u-u_{c}^{B} & v-\upsilon_{c}^{B} & w-w_{c}^{B} & p & q & r \end{array}\right]}^{T}$$

In this paper, the slowly varying ocean current is considered hence ${\dot{v}}_{r}\approx 0$. As a result, the dynamic equation motions of the AUV under the influence of the ocean current in the BF frame are:

(36) $$\begin{array}{l} \dot{\eta }=J(\eta )v_{r}+V_{c}^{E}\\ M\dot{v}+C_{RB}(v)v+C_{A}(v_{r})v_{r}+D(v_{r})v_{r}+G(\eta )=\tau \end{array}$$

## 3. Design the Heading Angle of the over-Actuated AUV Using Line of Sight Guidance

Due to the simple structure, light in computation, and easy implementation of the LOS algorithm, the LOS is currently applied more in autonomous navigation of marine vehicles [36,37]. Moreover, the working principle of the LOS method is to mimic the behavior of a helmsman, which drives the AUV towards a target point. Based on this, the AUV at any initial positions outside the desired route will converge and stay on the path. For these reasons, a modified LOS method is used in this paper to design the guidance system of the AUV. The desired heading angle of the AUV, which is used to define the yaw angle of the AUV, is not only related to the real-time position of the AUV but also the target waypoints. These waypoints have two components, $x_{k}$ and $y_{k}$, given by the operator. The AUV supposes a tracking target on the tracking path and then follows along the connecting line between the AUV position and the virtual tracking target. Once the AUV arrives at the desired path, the heading deviation is slowly decreased, and the desired path can be precisely tracked.

**Figure 3:**
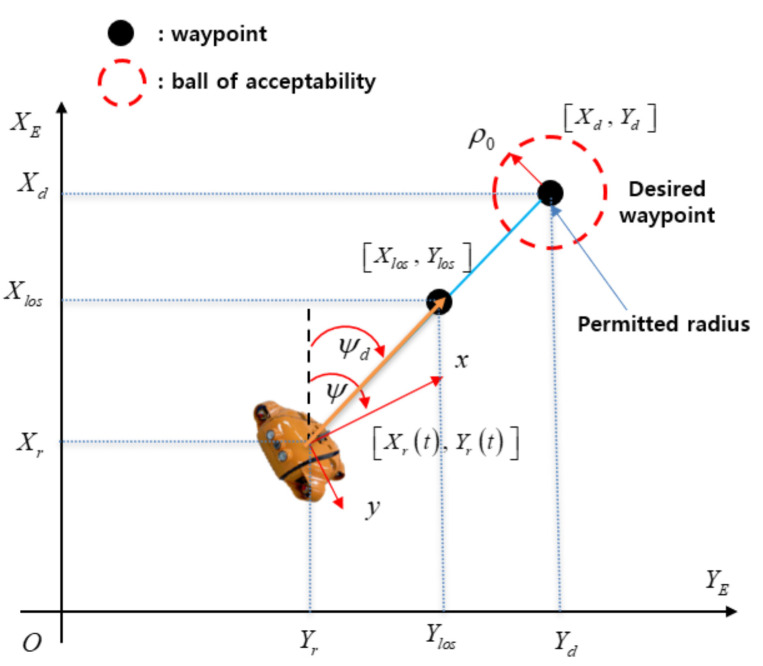
The proposed line of sight (LOS) method and the target waypoint.

According to Figure 3, the modified LOS position ($X_{los}(t)$, $Y_{los}(t)$) and the desired yaw angle $\psi_{d}(t)$ of the AUV can be calculated as:

(37) $$\sqrt{{\left(Y_{los}(t)-Y_{r}(t)\right)}^{2}+{\left(X_{los}(t)-X_{r}(t)\right)}^{2}}\leq \rho =nL_{pp}$$

(38) $$\frac{Y_{los}(t)-Y_{r}(t)}{X_{los}(t)-X_{r}(t)}=\frac{Y_{d}(t)-Y_{r}(t)}{X_{d}(t)-X_{r}(t)}=\mathrm{constant}$$

(39) $$\psi_{d}(t)=\tan^{-1}\left(\frac{Y_{los}(t)-Y_{r}(t)}{X_{los}(t)-X_{r}(t)}\right)$$

where, ρ is the safety radius that is set to the center of each waypoint, ($X_{k}$, $Y_{k}$) is the present waypoint, $L_{pp}$ is the length of the AUV; n is the positive value that combines with $L_{pp}$ to estimate the safety radius; ($X_{r}$, $Y_{r}$) and ($X_{d}$, $Y_{d}$) are two adjacent waypoints, in which one is the present waypoint or the actual position of the AUV that is estimated by global positioning system (GPS), and the other one is the desired waypoint. ψ(t) is the actual heading angle of the AUV measured by inertial measurement units (IMU) or compass sensor. ($x_{los}(t)$, $y_{los}(t)$) is the virtual tracking waypoint. The vector connecting between this virtual tracking waypoint and the actual position of the AUV is the “line of sight”. The angle between the “line of sight” and the north direction of the EF frame is the desired heading angle $\psi_{d}(t)$ for the heading control of the AUV is obtained by Equation (39).

## 4. Design of Dynamic Position Control for the Over-Actuated AUV

**Figure 4:**
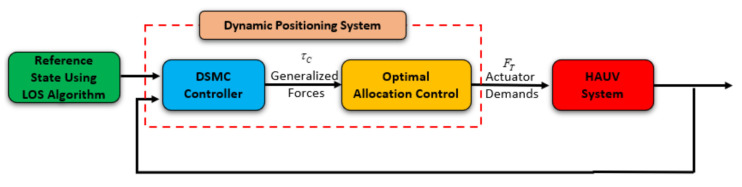
A dynamic positioning (DP) control system, including a motion control law and an allocation control module.

In this section, a DP control system has been used to the individual thruster again the environmental disturbance, for maintaining the position and the heading angle of the AUV. As can be observed in Figure 4, the entire proposed DP control is divided into two related parts, which are the motion control module and the allocation control module. In particular, the role of a motion control system that is designed by using a DSMC law is to generate the generalized control forces and moments. Meanwhile, the allocation control module decides how to distribute these generalized forces and moments to each individual thruster and how to optimize the energy consumption. Both mentioned parts of the suggested DP control are presented in the following sections.

### 4.1. Design of Motion Control for the over-Actuated AUV Using Dynamic Sliding Mode Controller

To overcome the effects of the ocean currents on the motions of the AUV, the robust DSMC is designed and presented in this section.

From Equation (36), the dynamics model of the AUV can be rewritten as:

(40) $$\begin{array}{l} \ddot{\eta }=\dot{J}(\psi )v+J(\psi )\dot{v}\\ =\dot{J}(\psi )v+J(\psi )M^{-1}\tau +J(\psi )M^{-1}\left[\tau_{d}-C(v)v-D(v)v-G(\eta )-f(\eta ,v)\right] \end{array}$$

Let $x_{1}=\eta$, $x_{2}=\dot{\eta }$, then the dynamics model of the AUV can be described as:

(41) $$\begin{array}{l} {\dot{x}}_{1}=x_{2}\\ {\dot{x}}_{2}=\dot{J}(\psi )v+J(\psi )M^{-1}\tau +J(\psi )M^{-1}\left[\tau_{d}-C(v)v-D(v)v-G(\eta )-f(\eta ,v)\right]\\ \;=\Omega \tau +\Omega \left(-C(v)v-D(v)v-G(\eta )\right)+\dot{J}(\psi )v+\Omega \left(\tau_{d}-f(\eta ,v)\right)\\ \;=\Omega u+\Phi (\eta ,v)+\Xi (\eta ,v,t) \end{array}$$

where, u=τ, $\Omega =J(\psi )M^{-1}$, $\Phi (\eta ,v)=\Omega \left(-C(v)v-D(v)v-G(\eta )\right)+\dot{J}(\psi )v$ represents the lumped nominal component, f(η,v) is the vector denotes model uncertainties and $\Xi (\eta ,v,t)=\Omega \left(\tau_{d}-f(\eta ,v)\right)$ denotes the lumped uncertainty.

Let $x_{d}=\eta_{d}$ represents the desired variables of the AUV in Equation (41), and $e=x_{d}-x_{1}$, $\dot{e}={\dot{x}}_{d}-x_{2}$ are the tracking error and its first derivative, respectively.

Using Equation (41), the second derivative of the tracking error, $\ddot{e}$, is expressed as:

(42) $$\begin{array}{l} \ddot{e}={\ddot{x}}_{d}-{\dot{x}}_{2}\\ ={\ddot{x}}_{d}-\Phi (x)-\Omega (x)u-\Xi (x,t) \end{array}$$

In this work, the proportional-integral-derivative (PID) sliding surface function is proposed as:

(43) $$s(t)=K_{P}e(t)+K_{I}\int_{0}^{t}e(\tau )d\tau +K_{D}\dot{e}(t)$$

where $K_{P},K_{I},K_{D}>0$ correspond to the proportional, integral, and derivative gains, respectively. The derivative of the sliding surface, $\dot{s}(t)$, is now obtained from Equation (43), as follows:

(44) $$\dot{s}(t)=K_{P}\dot{e}(t)+K_{I}e(t)+K_{D}\ddot{e}(t)$$

Let σ(t) be a new dynamic sliding surface given by:

(45) $$\sigma (t)=\dot{s}(t)+\lambda s(t)$$

where λ is a positive constant value. Obviously, if the value of σ=0, then the system in Equation (45) is asymptotically stable. Thus, $\underset{t\to \infty }{\lim}e(t)=0$, which indicates that the robust controller can be designed based on the new dynamic sliding surface σ(t).

Substituting Equations (42) and (44) into Equation (45), the dynamic sliding surface can be rewritten as:

(46) $$\sigma (t)=K_{P}\dot{e}(t)+K_{I}e(t)+K_{D}\left({\ddot{x}}_{d}-\Phi (x)-\Omega (x)u-\Xi (x,t)\right)+\lambda s(t)$$

Taking the first-order time derivative Equation (46) and combining the result with Equation (44) yields:

(47) $$\begin{array}{l} \dot{\sigma }(t)=K_{P}\ddot{e}(t)+K_{I}\dot{e}(t)+K_{D}\left({\dddot{x}}_{d}-\dot{\Phi }(x)-\dot{\Omega }(x)u-\Phi (x)\dot{u}-\dot{\Xi }(x,t)\right)+\lambda \left(K_{P}e(t)+K_{I}e(t)+K_{D}\ddot{e}(t)\right)\\ =K_{D}\left({\dddot{x}}_{d}-\dot{\Phi }(x)-\dot{\Omega }(x)u-\Phi (x)\dot{u}-\dot{\Xi }(x,t)\right)+\left(K_{P}+K_{D}\lambda \right)\ddot{e}(t)+\left(K_{I}+K_{P}\lambda \right)\dot{e}(t)+\lambda K_{I}e(t) \end{array}$$

From Equation (42), Equation (47) can be re-arranged as:

(48) $$\begin{array}{l} \dot{\sigma }(t)=K_{D}\left({\dddot{x}}_{d}-\dot{\Phi }(x)-\dot{\Omega }(x)u-\dot{\Xi }(x,t)\right)-K_{D}\Omega (x)\dot{u}+\left(K_{P}+K_{D}\lambda \right)\left({\ddot{x}}_{d}-\Phi (x)-\Omega (x)u-\Xi (x,t)\right)\\ +\left(K_{I}+K_{P}\lambda \right)\dot{e}(t)+\lambda K_{I}e(t) \end{array}$$

**Theorem** **1.** Let us assume that ∃ϑ, γ, $K_{s}\in R^{+}$ are constant values and always satisfy the below expression:

(49) $$\{\begin{array}{c} \left\|\tilde{\Xi }(\eta ,v,t)\right\|\leq \kappa ,\left\|\dot{\tilde{\Xi }}(\eta ,v,t)\right\|\leq \gamma \\ K_{D}\gamma +\left(K_{P}+K_{D}\lambda \right)\kappa \leq K_{s} \end{array}$$

The dynamic sliding surface, σ(t), asymptotically converges to zero if the comprehensive control law, $\dot{u}$, is chosen as:

(50) $$\dot{u}\left(t\right)=\frac{1}{K_{D}\Omega (x)}\left(\begin{array}{l} K_{D}\left({\dddot{x}}_{d}-\dot{\Phi }(x)-\dot{\Omega }(x)u-\dot{\hat{\Xi }}(x,t)\right)+\left(K_{P}+K_{D}\lambda \right)\left({\ddot{x}}_{d}-\Phi (x)-\Omega (x)u-\hat{\Xi }(x,t)\right)\\ +\left(K_{I}+K_{P}\lambda \right)\dot{e}(t)+\lambda K_{I}e(t)+\beta \sigma +K_{s}sign(\sigma ) \end{array}\right)$$

where β is a positive value.

**Proof** **of** **Theorem 1.** From Equations (48) and (50), the derivative of the dynamic sliding surface, $\dot{\sigma }(t)$, can be re-arranged as:

(51) $$\begin{array}{l} \Rightarrow \dot{\sigma }(t)=-K_{D}\left(\dot{\Xi }-\dot{\hat{\Xi }}\right)-\left(K_{P}+K_{D}\lambda \right)\left(\Xi -\hat{\Xi }\right)-\beta \sigma -K_{S}\mathrm{sgn}(\sigma )\\ =-K_{D}\dot{\tilde{\Xi }}-\left(K_{P}+K_{D}\lambda \right)\tilde{\Xi }-\beta \sigma -K_{S}\mathrm{sgn}(\sigma ) \end{array}$$

Consider the following Lyapunov function candidate:

(52) $$V_{1}=\frac{1}{2}\sigma^{2}(t)$$

Differentiating Equation (52) and combining the result with Equation (51), one obtains:

(53) $$\begin{array}{l} {\dot{V}}_{1}=\sigma \left(-K_{D}\dot{\tilde{\Xi }}-\left(K_{P}+K_{D}\lambda \right)\tilde{\Xi }-\beta \sigma -K_{S}\mathrm{sgn}(\sigma )\right)\\ =-\beta \sigma^{2}+\sigma \left(-K_{D}\dot{\tilde{\Xi }}-\left(K_{P}+K_{D}\lambda \right)\tilde{\Xi }\right)-K_{S}\left|\sigma \right| \end{array}$$

Using the condition in Equation (49), we have:

(54) $$\begin{array}{l} {\dot{V}}_{1}\leq -\beta \sigma^{2}+\left|\sigma \right|\left(K_{D}\gamma +\left(K_{P}+K_{D}\lambda \right)\kappa -K_{S}\right)\\ \leq -\beta \sigma^{2} \end{array}$$

As a result, the dynamic sliding surface, σ(t), asymptotically converges to zero according to the Lyapunov criterion. This proof is completed.

Hence, the proposed DSMC is designed as shown in Equation (50) with $K_{s}$ selected by Equation (49). By replacing the sign function with a saturating function expressed in Equation (55), the chattering problem in Equation (50) will be eliminated:

(55) $$sat(\frac{s}{\phi })=\{\begin{array}{c} \mathrm{sgn}(\frac{s}{\phi })\;\;\;\;if\;\;\left|\frac{s}{\phi }\right|>1\\ \frac{s}{\phi }\;\;\;\;otherwise \end{array}$$

Finally, the control law in Equation (50) now becomes:

(56) $$\dot{u}\left(t\right)=\frac{1}{K_{D}\Omega (x)}\left(\begin{array}{l} K_{D}\left({\dddot{x}}_{d}-\dot{\Phi }(x)-\dot{\Omega }(x)u-\dot{\hat{\Xi }}(x,t)\right)+\left(K_{P}+K_{D}\lambda \right)\left({\ddot{x}}_{d}-\Phi (x)-\Omega (x)u-\hat{\Xi }(x,t)\right)\\ +\left(K_{I}+K_{P}\lambda \right)\dot{e}(t)+\lambda K_{I}e(t)+\beta \sigma +K_{s}sat(\frac{s}{\phi }) \end{array}\right)$$

where ϕ>0 is the boundary layer thickness. □

### 4.2. Design of Motion Control for the Over-actuated AUV Using Dynamic Sliding Mode Controller

In this section, general methods of modeling and solving the allocation control problems are presented. Once the generalized forces and moments are defined by using the motion control, the allocation control module distributes suitable thruster forces to all thrusters of the AUV. Two optimal allocation control methods are designed and examined so as to achieve the DP maneuvering: one approach is the LS method without considering the thruster constraint, the other approach is the QP that considered the allocation control as a constrained optimization problem. The advantages and disadvantages of both methods will be analyzed below.

#### 4.2.1. Unconstrained Thrust Allocation Using Lagrange Multipliers

In this study, the AUV is installed with seven thrusters, and the thruster dynamics are ignored; therefore, the relationship between the vector of the thruster forces *F* and the vector of the generalized forces and moments $U_{v}$ can be expressed in the linear form as:

(57) $$U_{v}=LF$$

As mentioned in Section 2.4, the thruster configuration *L* is not a square matrix, which implies that the solution of Equation (57) is not unique. A common solution of offsetting for thruster redundancy of the AUV is to use the LS method. Now, we can generate a least-squares cost function in the form as

(58) $$\begin{array}{l} F^{*}=\arg\;\min\left(F^{T}WF\right)\\ subject\;\;to\;\;U_{v}=LF \end{array}$$

where *W* is a positive definite weighting matrix. The cost function in Equation (58) is considered as an energy consumption minimization problem.

To solve Equation (58), we choose the Lagrangian function as

(59) $$L(F,\lambda )=\frac{1}{2}F^{T}WF+\lambda^{T}(U_{v}-LF)$$

where λ denotes the Lagrange multiplier. By differentiating Equation (59) with respect to *F*, the Karush–Kuhn–Tucker (KKT) can be obtained:

(60) $$\frac{\partial L}{\partial F}=WF-L^{T}\lambda =0\Rightarrow F=W^{-1}L^{T}\lambda$$

Using Equation (57), we have:

(61) $$U_{v}=LW^{-1}L^{T}\lambda$$

Assuming that $LW^{-1}L^{T}$ is not singular, the optimal solution for the Lagrange multiplication is defined as:

(62) $$\lambda ={\left(LW^{-1}L^{T}\right)}^{-1}U_{v}$$

From Equation (60) and Equation (62), the vector F can be generated as follows:

(63) $$F=W^{-1}L^{T}{\left(LW^{1}L^{T}\right)}^{-1}U_{v}=L_{W}^{†}U_{v}$$

where $L_{W}^{†}=W^{-1}L^{T}{\left(LW^{1}L^{T}\right)}^{-1}$ is the generalized inverse.

#### 4.2.2. Constrained Thruster Allocation Using Quadratic Programming

In the previous discussions, we considered the optimal allocation control problem without the thruster saturation constraints. In practice, this assumption that is applied to the allocation control problem cannot provide sufficiently accurate approximations. For this, various methods to perform the thrust allocation with a constrained nonlinear model have been proposed [38,39]. In this section, the allocation control is achieved by using a QP strategy, which relates the minimization of a quadratic cost function subject to both equality and inequality constraints.

The QP is one of the most popular powerful approaches to thrust allocation. In the framework of the optimization, the thrust allocation problem can be modeled as:

(64) $$J=\underset{F,s}{\min}\left(F^{T}WF+s^{T}Qs\right)$$

subject to:

(65) $$\begin{array}{l} LF=U_{v}+s\\ F_{\min(i)}\leq F_{i}\leq F_{\max(i)}\;\;(i=1,\ldots ,k) \end{array}$$

where s is a slack variable and *W* and *Q* are the positive weighting parameter matrices of the thruster *F* and the slack variable s, respectively. Note that to obtain the feasible solution of the vector $U_{v}$, the slack variables should be close to zero by selecting the weight parameter Q much larger value than the weight parameter W in most cases.

By setting the new variables $z={\left(F^{T},s^{T}\right)}^{T}\in R^{k+n}$, and $x={\left(U_{v}^{T},F_{\min}^{T},F_{\max}^{T}\right)}^{T}\in R^{n+2k}$, Equations (64) and (65) can be re-arranged as follows:

(66) $$J=\underset{z}{\min}\left(z^{T}Kz\right)$$

Subject to

(67) $$\begin{array}{l} A_{1}z=C_{1}x\\ A_{2}z=C_{2}x \end{array}$$

All matrices *K*, $A_{1}$, $C_{1}$, $A_{2}$, $C_{2}$ in Equations (66) and (67) can be obtained, respectively, as follows:

(68) $$K=\left[\begin{array}{cc} W & 0_{k\times n}\\ 0_{n\times k} & Q \end{array}\right]$$

(69) $$A_{1}=\left[\begin{array}{cc} B & -I_{n\times n} \end{array}\right],C_{1}=\left[\begin{array}{cc} I_{n\times n} & 0_{n\times 2k} \end{array}\right]$$

(70) $$A_{2}=\left[\begin{array}{cc} -I_{k\times k} & 0_{k\times n}\\ I_{k\times k} & 0_{k\times n} \end{array}\right],C_{1}=\left[\begin{array}{ccc} 0_{k\times n} & -I_{k\times k} & 0_{n\times n}\\ I_{k\times k} & 0_{k\times n} & I_{k\times k} \end{array}\right]$$

As *W* and *Q* are the positive matrices, Equations (66) and (67) describe the convex QP, which indicates that the global solution of the optimal allocation control problem can be confirmed. With the advancement of computer technologies, the problem formulation can be solved using a QP solver or mathematical optimization software.

## 5. Simulation Results and Discussion

**Figure 5:**
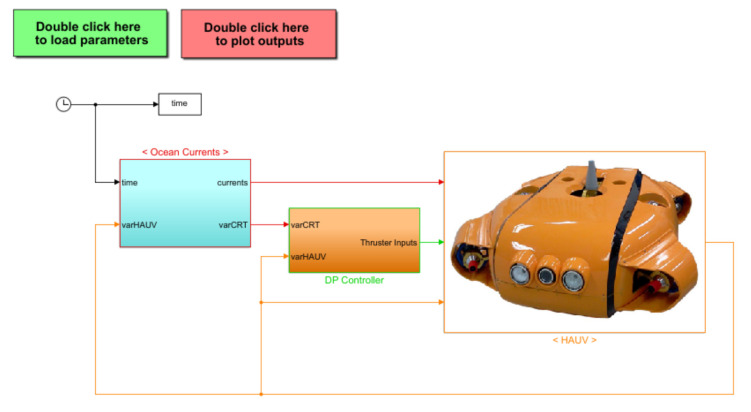
Simulation program.

Because the dynamic model of an AUV in the presence of model uncertainties and ocean current disturbances is complex and unstable, it is challenging to simulate the behaviors of the AUV under the input of a new controller. In this section, the numerical simulations based on MATLAB/Simulink environments are performed to demonstrate the performance of the suggested controller, as shown in Figure 5. The crucial parameters of the simulated AUV system are shown in Table 1 and Table 2, respectively.

The following two simulations have been performed as:

- **Simulation 1:** The effects of the ocean currents on the AUV motions;
- **Simulation 2**: The position stabilization control of the AUV in six-DOFs in the presence of the ocean currents and the model uncertainties.

### 5.1. Simulate the Effects of Ocean Currents on the over-Actuated AUV

**Figure 6:**
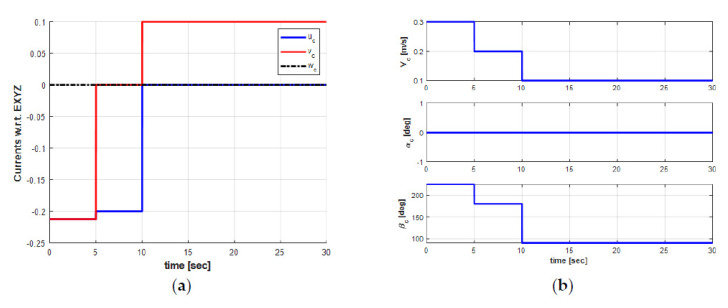
Ocean current form: (**a**) ocean current vector w.r.t Earth−fixed coordinate; (**b**) ocean current parameters.

Because the ocean currents have significant effects on the tracking performance of AUV, it is necessary to consider it when modeling and designing new controllers for AUV. Unfortunately, the real ocean current model is very complex and therefore, it is difficult to model the exact real ocean current in a simulation environment. In this paper, we assume that the ocean current is irrational and varies very slowly with respect to time, as discussed in Section 2.5. Particularly, using the first Gauss–Markov process, the ocean current can be modeled in 3D Cartesian space with an average speed ($V_{c}$), and two orientation angles, i.e., the angle of attack ($\beta_{c}$), and the sideslip angle ($\alpha_{c}$) in the EF frame as shown in Figure 6b.

Based on the selected parameters of the ocean current, a numerical simulation is carried out to understand the influence of the ocean currents on the dynamic behaviors of the AUV. The dynamic responses of the AUV to the ocean current effects while doing the turning motion are observed in this simulation. To achieve a pure turning motion of the AUV, we set the different thrust forces on the four horizontal thrusters as $T_{1}=10\;N$, $T_{2}=11\;N$, $T_{3}=-10\;N$, and $T_{4}=-11\;N$, respectively, while all three vertical thrusters $T_{5}$, $T_{6}$, $T_{7}$ are equal to 0 N. The duration and the sampling time of this simulation are 30 s and 0.01 s, respectively.

**Figure 7:**
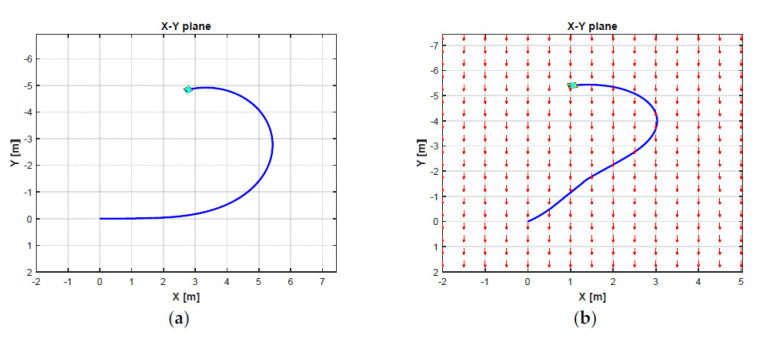
2D trajectories of the AUV in turning motion: (**a**) without current effects; (**b**) with current effects.

**Figure 8:**
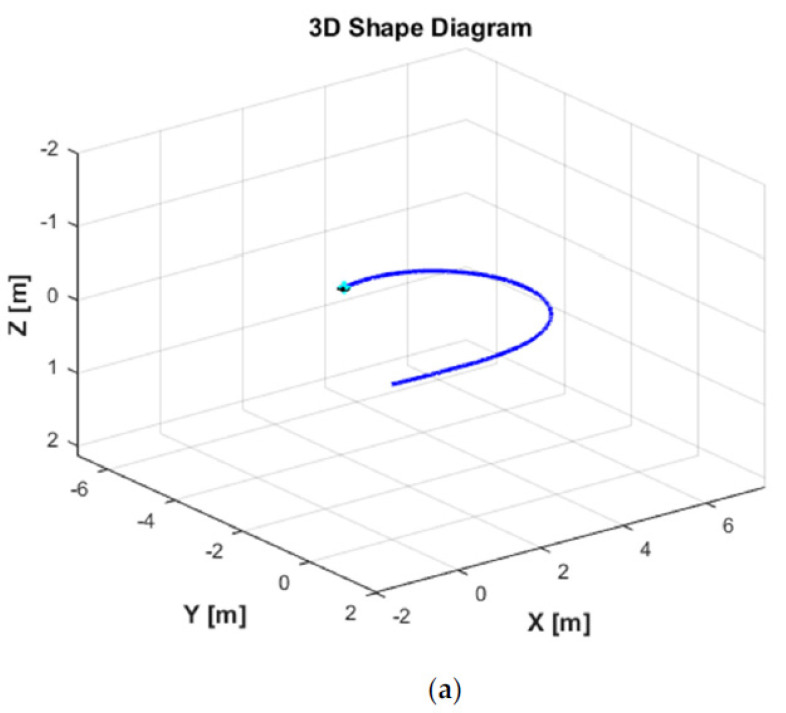

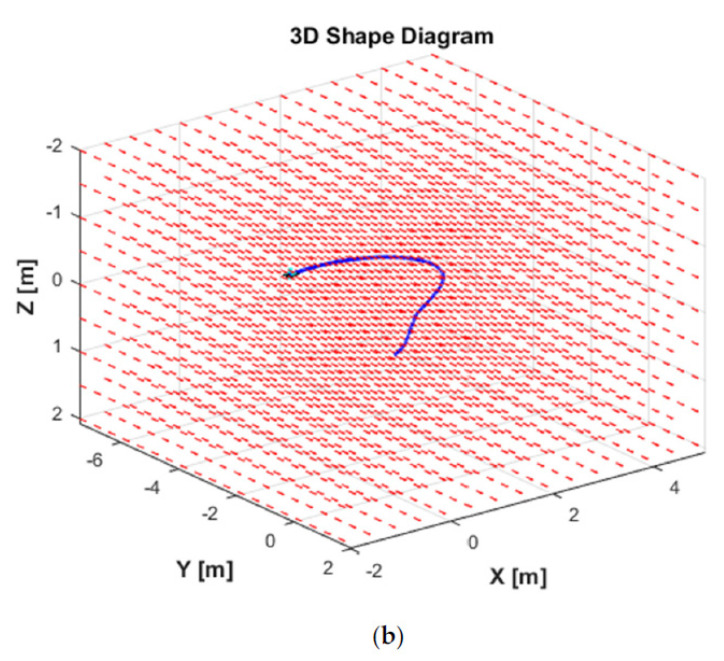
3D trajectories of the AUV in turning motion: (**a**) without current effects; (**b**) with current effects.

Figure 6 shows the ocean current form with its three parameters in the EF frame. As can be seen in Figure 6b, the ocean current flows in the direction between South and West directions with the following parameters: $V_{c}=0.3$ m/s, $\alpha_{c}=0$ degree, and $\beta_{c}=225$ degrees from t = 0 s to t = 5 s. Then, the ocean current unexpectedly changes its direction to the South direction with the changing parameters, i.e., $V_{c}=0.2$ m/s, $\alpha_{c}=0$ degree, and $\beta_{c}=180$ degrees from t > 5 s to t = 10 s. Finally, the ocean current suddenly varies its direction to the East direction, and the parameters are the following: $V_{c}=0.1$ m/s, $\alpha_{c}=0$ degree, and $\beta_{c}=90$ degrees from t > 10 s to the end of the simulation time t = 30 s. In addition, the ocean current vector in the EF frame during the simulation is shown in Figure 6a. Under these ocean current configurations, the 2D trajectory behavior of the AUV during the turning motion can be observed in Figure 7. Meanwhile, the significant effect of the ocean currents on the turning motion of the AUV can be observed in the 3D trajectory shown in Figure 8.

**Figure 9:**
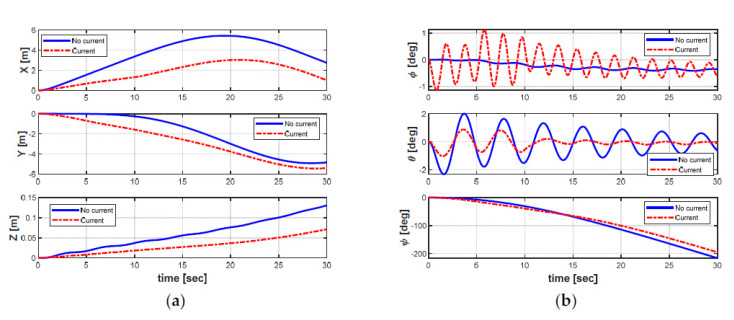
AUV dynamic behaviors: (**a**) position; (**b**) Euler angles.

**Figure 10:**
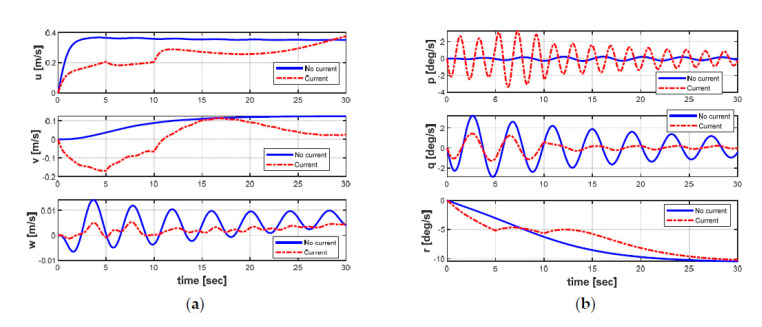
AUV dynamic behaviors: (**a**) linear velocities; (**b**) angular velocities.

Furthermore, the effects of the ocean currents on the position, orientation, and velocities of the AUV can be obviously observed in Figure 9 and Figure 10, respectively. From Figure 9 and Figure 10, it can be seen that all states of the AUV are seriously affected by the variants of ocean currents during the turning motion. Especially, the roll and pitch motions of the AUV appeared to be significantly oscillatory. In general, the perturbation can be observed at t = 5 s and t = 10 s in Figure 10 when the ocean current suddenly changes its directions and the average speeds after every five seconds time (from t = 0 s to t = 10 s).

### 5.2. Dynamic Position of the over-Actuated AUV in Six-DOF

The influences of the ocean currents on the motion of the AUV have been observed and analyzed through the numerical simulation in Section 5.1. In order to perform the position stabilization control of the AUV under the ocean current effects and the model uncertainties, a new control method, which includes two modules: the motion control and the optimal allocation control, is designed. The effectiveness of the suggested control will be demonstrated via the simulation results in this section.

In this simulation, we assume that the model parameters are disturbed from their actual values by 30%. Meanwhile, the irrotational ocean current model effects on the AUV are generated by the first-order Gauss–Markov processes, and the variation of its parameters $V_{c}$, $\alpha_{c}$, and $\beta_{c}$ are defined as the same as in Section 5.1.

To prove the ability to provide optimal control efforts of the suggested allocation control strategies, a numerical simulation is performed to compare the performance between “unconstrained thrust allocation” and “constrained thrust allocation” cases in this section. The setting conditions for the simulation of both methods can be explained as below:

**Unconstrained Thrust Allocation:** In this case, the unconstrained thrust allocation is applied using the standard damped inverse, i.e., the LS method. To do so, a high virtual constraint on the individual thruster force $u_{i}$ is applied to guarantee that the saturation limit on each thruster will never occur. Thus, these constraints are bounded to $u_{\max,i}=1000N$ and $u_{\min,i}=-1000N$ in this simulation.

**Constrained Thrust Allocation:** The constrained thrust allocation is considered using the QP method. In this case, the generalized forces are subject to the saturation constraints according to the physical limitation of the individual thruster. For this, the saturation constraints are set to $u_{\max,i}=100N$ and $u_{\min,i}=-100N$ for the seven thrusters of the AUV.

For simulation purposes, the AUV is requested to stay at the desired point $\left[x_{d},y_{d},z_{d},\phi_{d},\theta_{d},\psi_{d}\right]=\left[3,2,10,0,0,LOS\right]$ from the initial point $\left[x_{0},y_{0},z_{0},\phi_{0},\theta_{0},\psi_{0}\right]=\left[0,0,0,0,0,0\right]$, where the desired heading angle is generated by the LOS under the changing direction of the ocean current in the simulation. Furthermore, the initial velocity of the AUV is set to be [u,v,w,p,q,r]=[0.3,0,0,0,0,0]. The behavior of the ocean current effects acting on the AUV can be observed again in Figure 6. It can be seen that, at the first stage (0≤t≤5 [s]), the setting direction of the ocean current is between the South and West directions. Meanwhile, at the second stage (5<t≤10 [s]), the ocean current $u_{c}$ flows towards the x-direction, and at the third stage (10<t≤30 [s]), the ocean current $v_{c}$ flows towards the y-direction.

**Figure 11:**
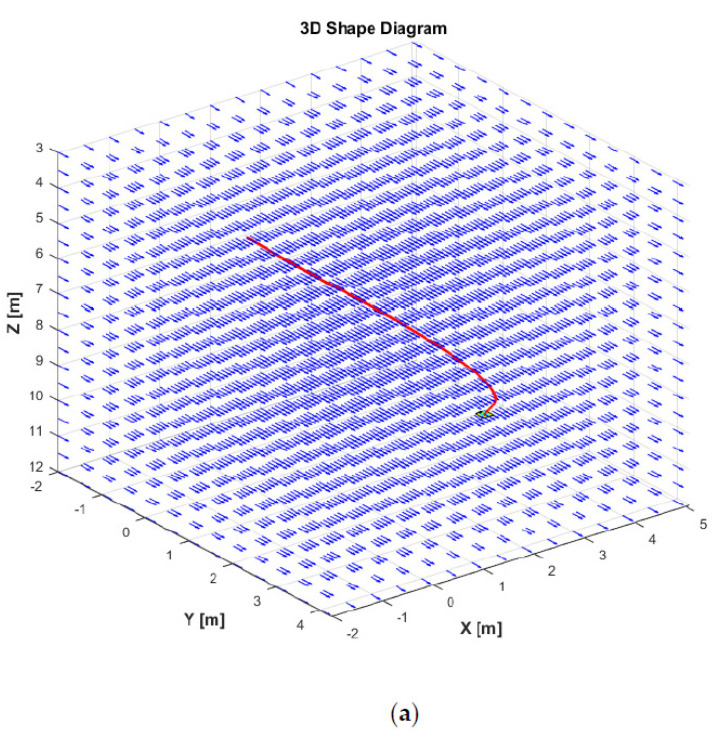

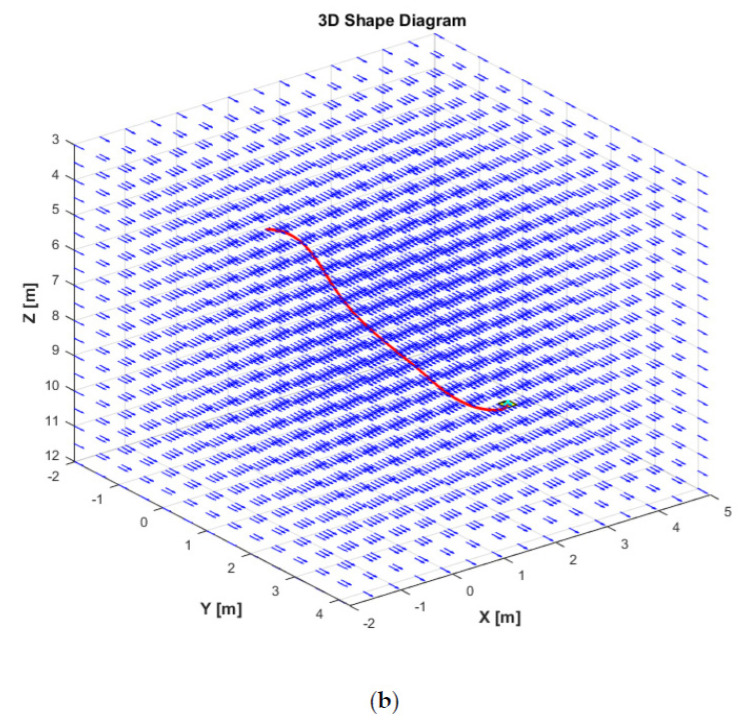
3D trajectories of the AUV: (**a**) using DSMC + LS method; (**b**) using DSMC + QP method.

Figure 11 displays the trajectories of the AUV in a 3D plane for two different simulation scenarios: one using the DSMC and the LS method (DSMC + LS), and the other applying the DSMC and the QP method (DSMC + QP). As shown in Figure 11, the trajectory tracking results of the AUV are superimposed with the ocean currents vector. It can also be seen that the AUV starts from the initial condition, then is required to reach and stay at a certain position (x, y, z, ϕ, θ) = (3,2,10,0,0) with the changing yaw angle ψ during the simulation.

**Figure 12:**
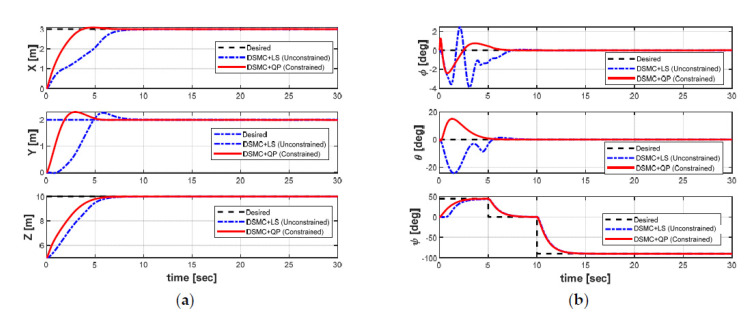
Position and orientation of the AUV in both cases: (**a**) position; (**b**) Euler angles.

**Figure 13:**
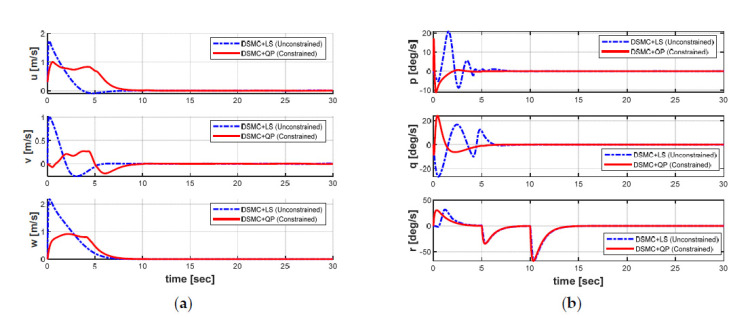
Velocities of the AUV in both cases: (**a**) linear velocities; (**b**) angular velocities.

Figure 12 shows the variation of the linear position and Euler angles of the AUV in the presence of the ocean currents under both controllers, whereas the behaviors of the velocities of the AUV in both cases are shown in Figure 13. The differences between both controllers can be clearly observed in Figure 12 and Figure 13. Simulation results indicate that both controllers for the position stabilization can force the AUV to reach the target position with the desired heading angle. In addition, the velocities of the AUV can be convergent to zero under the controllers. However, the behavior of the DSMC + QP controller is obviously more stable than that of the DSMC + LS controller in the presence of the model uncertainties and the ocean currents with shorter convergence time, smaller overshoot, and achieve significantly higher accuracy. Furthermore, the position error in the roll and pitch motions of the AUV has some fluctuations in both controllers, but the fluctuation of the DSMC + QP method is much smaller. These results proved that the DSMC + QP method designed in this paper provide higher robustness and effectiveness, which verifies that the proposed DSMC + QP strategy may be available for the position stabilization control of the AUV despite the existence of the uncertainties model and the ocean currents.

In the case of implementing the DP under the effects of the ocean currents, the AUV rotates its heading angle against the environmental disturbance by changing the yaw angle set-point, which is acquired from the ocean current estimation and thrust usage of the DP system. In Figure 12b, the yaw angle is operated by the DSMC to follow the desired yaw angle generated by the LOS method. In this case, the target heading angles are 45, 0, and −90 degrees. As shown in Figure 12b, the AUV can reach the target heading angle in the vicinity and stabilizes quickly, thus, achieves successfully heading angle control.

**Figure 14:**
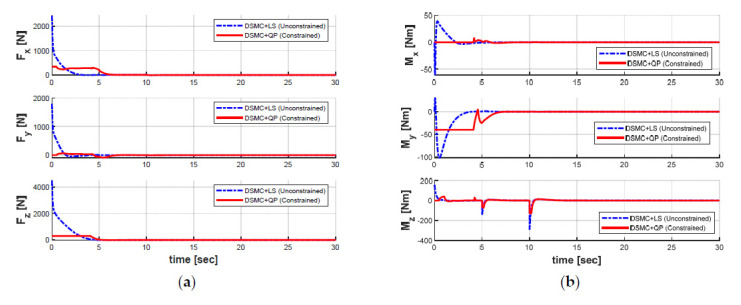
Control inputs of controller in both cases: (**a**) forces; (**b**) moments.

**Figure 15:**
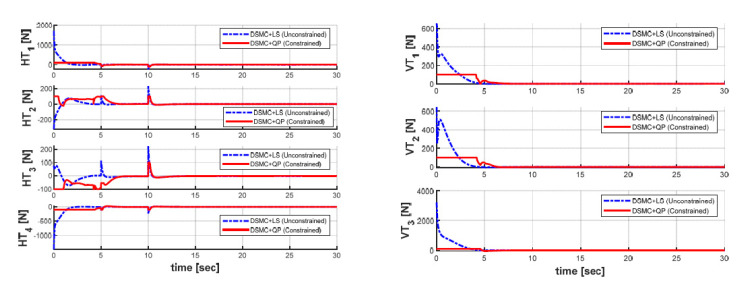
Seven thruster forces in both cases: (**a**) four horizontal thrusters; (**b**) three vertical thrusters.

In order to keep the target position while maintaining the desired heading angle, the generalized forces and moments obtained through the DSMC module for two optimal LS and QP methods are shown in Figure 14, whereas the response curves of the seven thrust forces obtained through the AC module for both cases are shown in Figure 15. The differences in the generalized forces and moments and the individual thruster forces of all the thrusters between the LS and the QP algorithms are clearly observed based on Figure 14 and Figure 15.

We observe that the generalized forces and thrust forces for both cases are raised seriously at the time of 5 s and 10 s to compensate for the ocean current disturbance. Especially, the torques in the yaw motion of the AUV are enhanced in order to counteract the excessive ocean currents. Compared with the QP algorithm, the LS algorithm yields greater thrust forces to all thrusters; this is because the LS method cannot generate the optimal solutions with unlimited thrust capabilities. It can also be seen from Figure 15 that the thrust generated in each thruster using the QP is controlled within the saturation constraints ± 100 N of the predefined thrust according to the real technical specifications of each thruster. Overall, it can be concluded that the QP control allocation algorithm proposed in this paper is more efficient in optimizing the energy consumed than the LS control allocation.

## 6. Conclusions

In this paper, the three-dimensional DP control problem of an over-actuated AUV with seven thrusters under the ocean current disturbances and the uncertainties model is addressed. During the DP control action, the AUV is requested to keep the linear position and the yaw angle with respect to a fixed reference point. This paper proposes a DP control system with two integrated modules, namely the motion control law and the control allocation. First, to improve the system robustness, a robust DSMC is developed for the motion control of the AUV under the assumption that the bounds of the external disturbance are known. Next, to handle the unconstrained and constrained allocation control problem, two strategies are designed and compared for the DP control system of the over-actuated AUV, i.e., the LS and QP methods. The stability of the proposed controller is then proved using the Lyapunov theorem. Finally, the simulation results are conducted to illustrate how the motion control law and allocation control modules interact to obtain the desired trajectory tracking performance while minimizing the power consumption of the seven thrusters on the over-actuated AUV. The simulation results show that the QP algorithm can significantly improve the performance of the DP control system, and it is able to solve the DP problem rapidly and precisely with the thrust force constraints.

## Figures and Tables

**Table 1:** Hydrodynamic coefficients.

Parameters	Units	Value	Description
$X_{\dot{u}}$	*kg*	−29	Added mass
$X_{u}$	*kg/s*	−72	Linear damping
$X_{u\left|u\right|}$	*kg/m*	−227.18	Axial drag
$Y_{\dot{v}}$	*kg*	−30	Added mass
$Y_{\dot{r}}$	*kg.m*	1.93	Added mass
$Y_{v}$	*kg/s*	−77	Linear damping
$Y_{v\left|v\right|}$	*kg/m*	−405.41	Crossflow drag
$Z_{\dot{w}}$	*kg*	−90	Added mass
$Z_{\dot{q}}$	*kg.m*	−1.93	Added mass
$Z_{w}$	*kg/s*	−95	Linear damping
$Z_{w\left|w\right|}$	*kg/m*	−478.03	Crossflow drag
$K_{\dot{p}}$	*kg.m*	−5.2	Added mass
$K_{p}$	*kg.m/s*	−40	Linear damping
$K_{p\left|p\right|}$	*kg.m*	−3.212	Rolling drag
$M_{\dot{w}}$	*kg*	−1.93	Added mass
$M_{\dot{q}}$	*kg.m*	−7.2	Added mass
$M_{q}$	*kg.m/s*	−30	Linear damping
$M_{q\left|q\right|}$	*kg.m*	−14.002	Crossflow drag
$N_{\dot{v}}$	*kg*	1.93	Added mass
$N_{\dot{r}}$	*kg.m*	−3.3	Added mass
$N_{r}$	*kg.m/s*	−30	Linear damping
$N_{r\left|r\right|}$	*kg.m*	−12.937	Crossflow drag

**Table 2:** The parameters for the simulation.

Properties	Units	Symbols	Values
AUV Parameters			
Dimension of the AUV	*mm*	L×B×H	560 × 750 × 280
Weight of the AUV	*kg*	m	80
Center of gravity	*m*	$X_{G}$	(0,0,−0.06)
Center of buoyancy	*m*	$X_{B}$	(0,0,0)
Inertia tensor in the x-axis	*kg.m^2^*	$I_{xx}$	6.9
Inertia tensor in the y-axis	*kg.m^2^*	$I_{yy}$	26.1
Inertia tensor in the z-axis	*kg.m^2^*	$I_{zz}$	23.2
**Initial Values and Desired Trajectory**			
Initial position of the AUV	*m/degree*	$\left[X_{0},Y_{0},Z_{0},\phi_{0},\theta_{0},\psi_{0}\right]$	[0;0;0;0;0;0]
Initial velocity of the AUV	*m/degree*	$\left[u_{0},v_{0},w_{0},p_{0},q_{0},r_{0}\right]$	[0.3;0;0;0;0;0]
Desired point of the AUV	*m/degree*	$\left[X_{d},Y_{d},Z_{d},\phi_{d},\theta_{d},\psi_{d}\right]$	[3;2;10;0;0;LOS]
**Parameter of the DSMC Controller**			
Parameter 1: $K_{P}$	*-*	$\left[K_{Px},K_{Py},K_{Pz},K_{P\phi },K_{P\theta },K_{P\psi }\right]$	[7;7;7;7;7;9]
Parameter 2: $K_{I}$	*-*	$\left[K_{Ix},K_{Iy},K_{Iz},K_{I\phi },K_{I\theta },K_{I\psi }\right]$	[0.06;0.06;0.06;0.06;0.06;0.06]
Parameter 3: $K_{D}$	*-*	$\left[K_{Dx},K_{Dy},K_{Dz},K_{D\phi },K_{D\theta },K_{D\psi }\right]$	[0.5;0.5;0.5;0.5;0.5;0.5]
Parameter 4: $K_{S}$	*-*	$\left[K_{Sx},K_{Sy},K_{Sz},K_{S\phi },K_{S\theta },K_{S\psi }\right]$	[8;8;8;8;8;10]

## Data Availability

Not Applicable.
